# Supplementary material for: Gastrin-releasing peptide signaling in the nucleus accumbens medial shell regulates neuronal excitability and motivation
Source: Nat Commun. 2025 Oct 21;16:9314. doi: 10.1038/s41467-025-64373-3 (PMC12540653; doi:10.1038/s41467-025-64373-3)
Supplement: Supplementary file 1 — Supplementary Information [file 41467_2025_64373_MOESM1_ESM.pdf]

Supplementary Information for:

Gastrin-releasing peptide signaling in the nucleus accumbens medial shell regulates neuronal excitability and motivation

Erin E. Aisenberg[1,2], Thomas L. Li[2,3], Hongli Wang[2,3], Atehsa A. Sahagun[1,2], Emilie M. Tu[3], and Helen S. Bateup[1,2,3*]

[1]Helen Wills Neuroscience Institute, University of California, Berkeley, Berkeley, CA, USA

[2]Department of Neuroscience, University of California, Berkeley, Berkeley, CA USA

[3]Department of Molecular and Cell Biology, University of California, Berkeley, Berkeley, CA USA

*Correspondence to: bateup@berkeley.edu

Contents: Supplementary Figures 1-17

Supplementary Figure 1

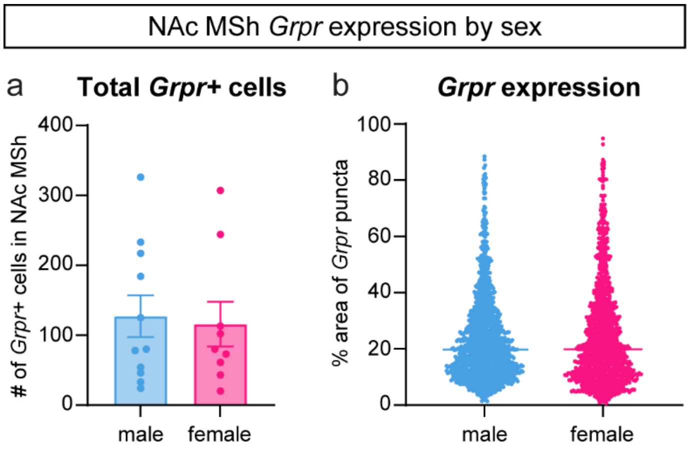

Supplementary Fig. 1: NAc MSh *Grpr* expression does not differ by sex.

(a) Mean ± SEM total number of *Grpr*+ cells in the NAc MSh by sex. n=11 male (blue) and 9 female (pink) mice; p=0.8973, two-tailed Mann-Whitney test. Dots represent values for individual mice. **(b)** Scatterplots showing the percent nuclear area covered by *Grpr* mRNA puncta in *Grpr*-expressing NAc MSh neurons for male and female mice. Lines represent the median and dots represent individual neurons (n=1339 cells from 11 male mice and n=1089 cells from 9 female mice; p=0.1097, unpaired two-tailed t-test). Source data are provided as a Source Data file. Related to Figure 1.

Supplementary Figure 2

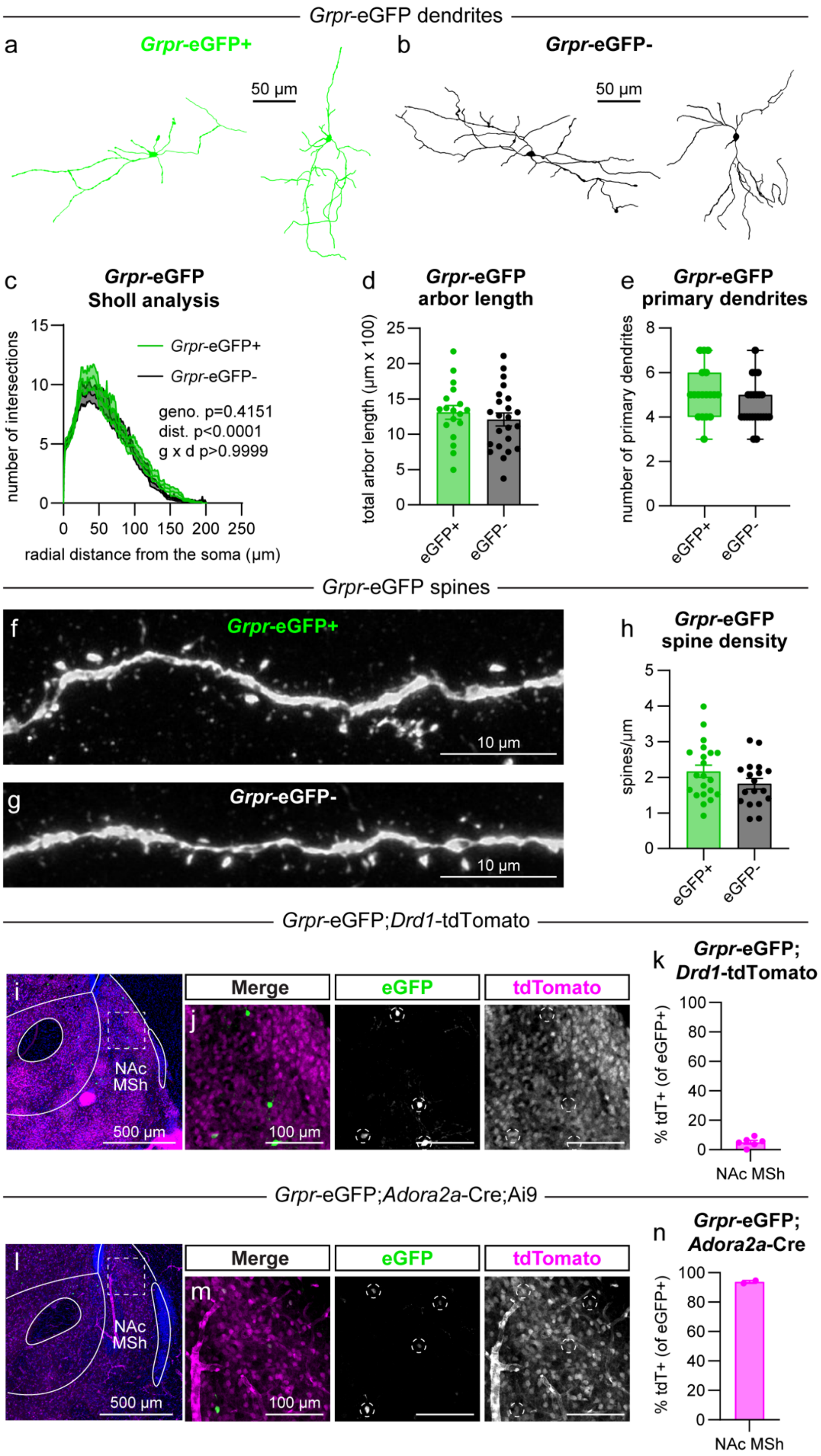

Supplementary Fig. 2: GRPR-expressing neurons in the NAc MSh are morphologically similar to neighboring neurons.

(a-b) Reconstructions of the dendrites and cell body of NAc MSh *Grpr*-eGFP+ neurons (**a,** representative of 18 neurons) and *Grpr*-eGFP- neurons (**b,** representative of 23 neurons). **(c)** Sholl analysis of NAc MSh neuron dendritic arbors. Dark colored lines are the mean, lighter color shading represents the SEM. *Grpr*-eGFP+ in green: n=18 neurons from 8 mice, *Grpr*-eGFP- in black: n=23 neurons from 14 mice. Two-way ANOVA p-values are shown. **(d)** Mean ± SEM total dendritic arbor length per cell. n is the same as for panel **c** (p=0.4452, Welch's two-tailed t-test). **(e)** Box plots of the number of primary dendrites per neuron for each genotype (boxes represent the interquartile range (25-75%), lines denote the median, whiskers represent mix to max values). n is the same as for panel **c** (p=0.1151, Welch's two-tailed t-test). **(f-g)** Representative images of mCherry-expressing dendrites from a *Grpr*-eGFP+ neuron (**f**, representative of 21 neurons) and *Grpr*-eGFP- neuron (**g**, representative of 18 neurons) in the NAc MSh. **(h)** Mean ± SEM spine density in *Grpr*-eGFP+ neurons vs *Grpr*-eGFP- neurons (*Grpr*-eGFP+ in green: n=21 neurons from 11 mice, *Grpr*-eGFP- in black: n=18 neurons from 9 mice; p=0.1357, Welch's two-tailed t-test). **(i)** Representative coronal brain section from a *Grpr*-eGFP;*Drd1*-tdTomato mouse showing the NAc MSh (representative of 6 mice). **(j)** Zoom-in of the boxed region showing lack of co-localization between eGFP and tdTomato. **(k)** Mean ± SEM percentage of NAc MSh eGFP+ cells that are tdTomato+ in *Grpr*-eGFP;*Drd1*-tdTomato mice (n=6 mice). **(l)** Representative coronal brain section from a *Grpr*-eGFP;*Adora2a*-Cre;Ai9 mouse showing the NAc MSh (representative of 2 mice). **(m)** Zoomed-in images of the boxed region showing that eGFP+ cells co-express tdTomato. **(n)** Mean ± SEM percentage of NAc MSh eGFP+ cells that are tdTomato+ in *Grpr*-eGFP;*Adora2a*-Cre;Ai9 mice (n=2 mice). For panels **d**, **e**, **h**, **k** and **n**, dots represent values for individual mice. Source data are provided as a Source Data file. Related to Figure 1.

Supplementary Figure 3

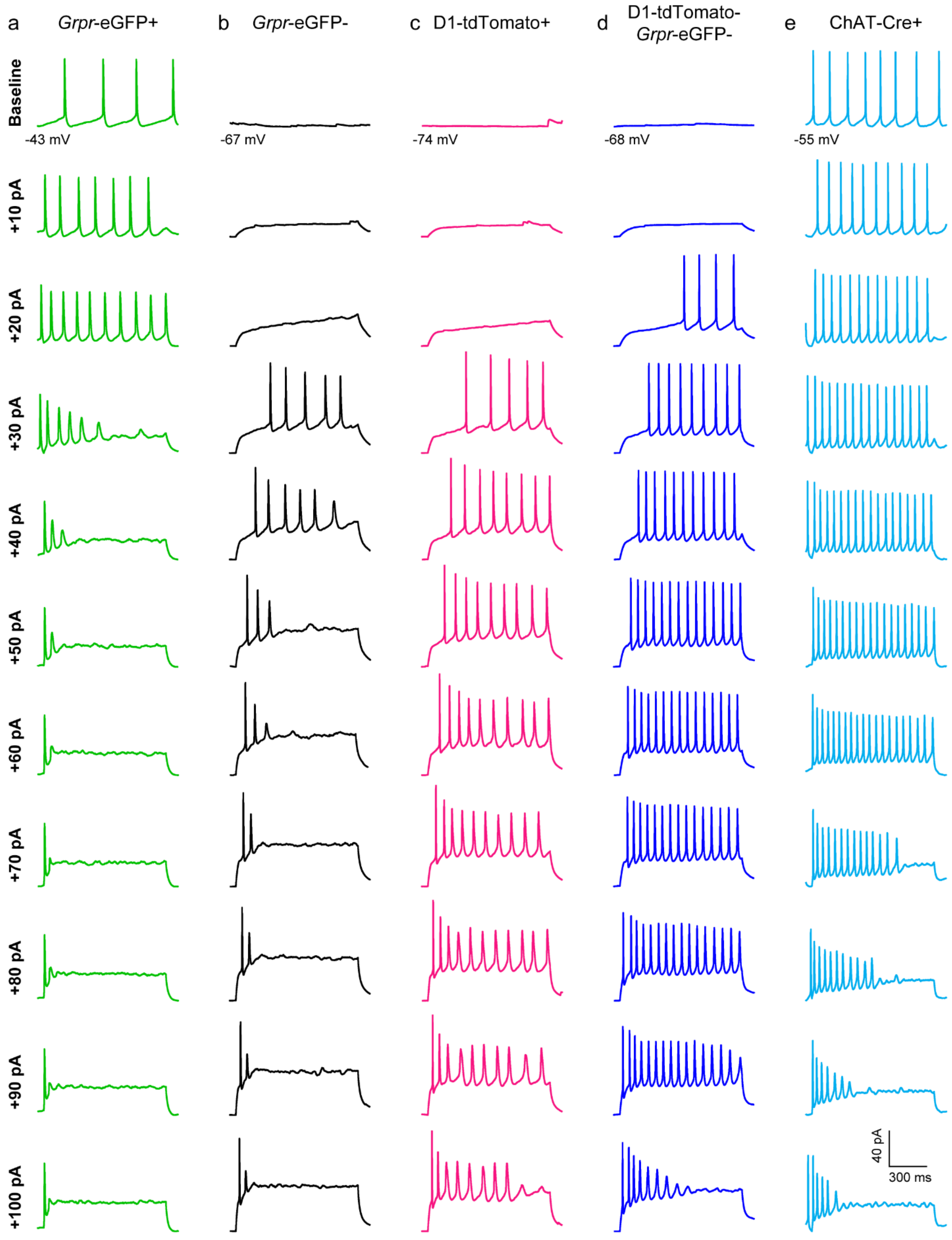

Supplementary Fig. 3: Example traces in response to positive current steps for different NAc MSh cell types.

(a-e) Example traces elicited by the indicated current steps for a representative *Grpr*-eGFP+ neuron (**a**, green, representative of 23 neurons), *Grpr*-eGFP- neuron (**b**, black, representative of 21 neurons), D1-tdTomato+ neuron (**c,** magenta, representative of 31 neurons), D1-tdTomato-;*Grpr*-eGFP- neuron (**d**, blue, representative of 27 neurons), and ChAT-Cre+ neuron (**e**, cyan, representative of 34 neurons). Related to Figure 1.

Supplementary Figure 4

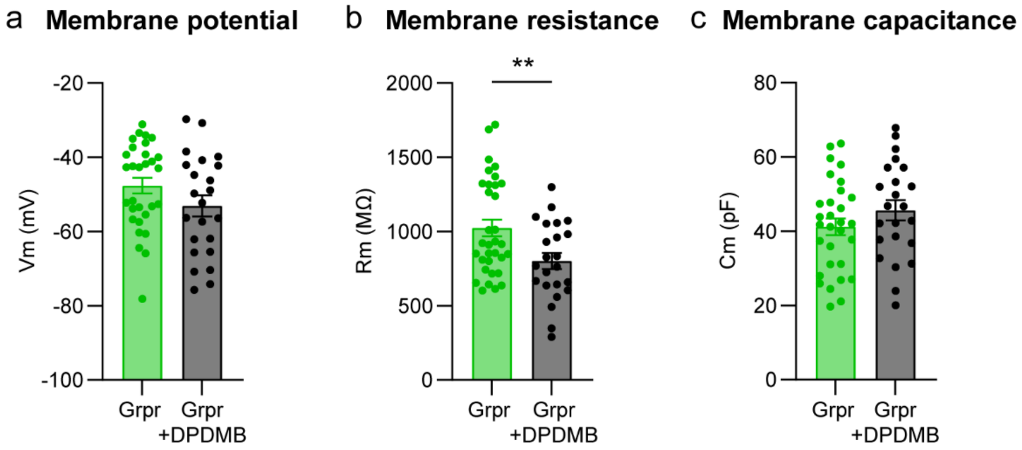

Supplementary Fig. 4: DPDMB decreases the membrane resistance of GRPR-expressing neurons in the NAc MSh.

(a) Mean ± SEM resting membrane potential of *Grpr*-eGFP+ NAc MSh neurons. *Grpr*-eGFP+ no DPDMB in green: n=30 cells from 15 mice and *Grpr*-eGFP+ with 1 μM DPDMB in black n=23 cells from 9 mice for all panels (Welch's two-tailed t-test, p=0.1288). **(b)** Mean ± SEM membrane resistance of *Grpr*-eGFP+ NAc MSh neurons with (black) and without (green) DPDMB (Welch's two-tailed t-test, **p=0.0066). **(c)** Mean ± SEM membrane capacitance of *Grpr*-eGFP+ NAc MSh neurons with (black) and without (green) DPDMB (Welch's two-tailed t-test, p=0.2199). For all bar graphs, dots represent values for individual cells. Source data are provided as a Source Data file. Related to Figure 1.

Supplementary Figure 5

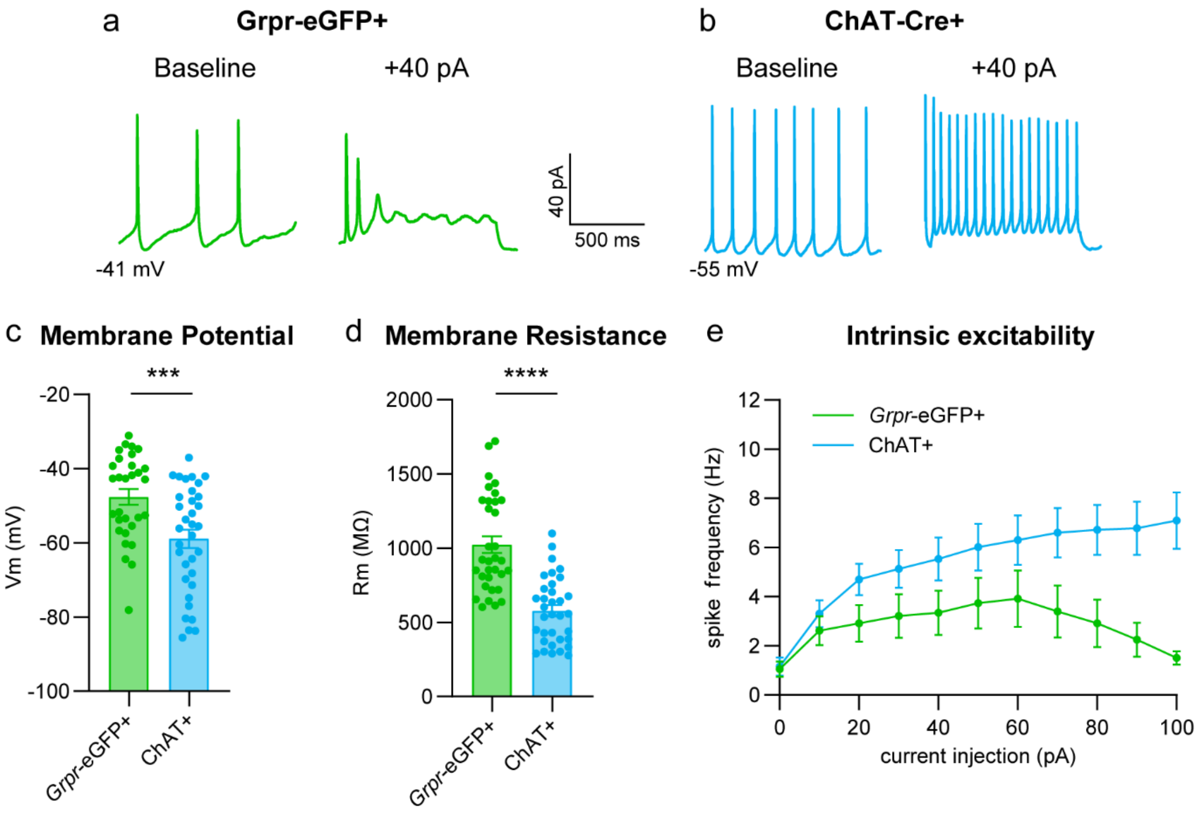

Supplementary Fig. 5: GRPR-expressing neurons in the NAc MSh have distinct electrophysiological properties from cholinergic interneurons.

(a-b) Representative traces from a *Grpr*-eGFP+ neuron (**a**, representative of 23 neurons) and *Chat*-Cre+ neuron (**b**, representative of 34 neurons) in the NAc MSh recorded at baseline (left) and following +40 pA current injection (right). **(c)** Mean ± SEM resting membrane potential of NAc MSh neurons. *Grpr*-eGFP+ cells in green: n=30 cells from 15 mice and *Chat*-Cre+ cells in light blue: n=34 cells from 7 mice (Welch's two-tailed t-test, ***p=0.0009). **(d)** Mean ± SEM membrane resistance of NAc MSh neurons. *Grpr*-eGFP+ in green: n=33 cells from 15 mice and *Chat*-Cre+ in light blue: n=34 cells from 7 mice. (Welch's two-tailed t-test, ***p<0.0001). **(e)** Input-output curves showing the mean ± SEM firing frequency of NAc MSh neurons in response to positive current steps of increasing amplitude. *Grpr*-eGFP+ in green: n=23 cells from 10 mice and *Chat*-Cre+ in light blue: n=34 cells from 7 mice. (Two-way repeated measures ANOVA, current injection x cell type ***p=0.0008, current injection **p=0.0010, cell type **p=0.0079). Data for *Grpr*-eGFP+ cells are duplicated from Fig. 1 for comparison. For all bar graphs, dots represent values for individual cells. Source data are provided as a Source Data file. Related to Figure 1.

Supplementary Figure 6

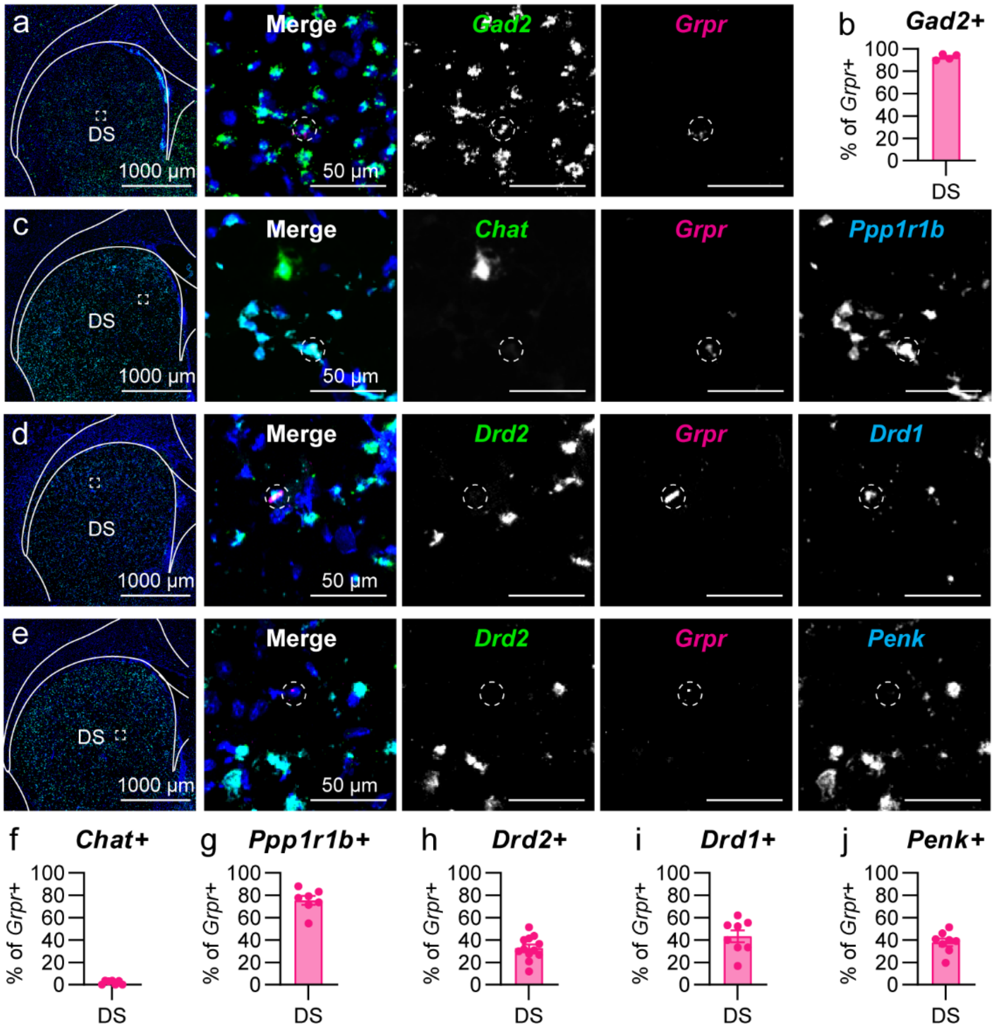

Supplementary Fig. 6: *Grpr* is expressed in SPNs in the dorsal striatum.

(a) Representative image of the dorsal striatum (DS, representative of 4 mice). FISH for *Gad2* in green and *Grpr* in magenta. Nuclei are labeled with DAPI in blue. Right panels show zoomed-in images of the boxed region. Dashed circle outlines *Grpr*+ cell. **(b)** Mean ± SEM percentage of *Grpr*+ cells in the DS that are *Gad2*+ in WT mice. (n=4 mice). **(c-e)** Representative images of the DS. Nuclei labeled with DAPI in blue. Dashed circles outline *Grpr*+ cells. **(c)** FISH for *Chat* in green, *Grpr* in magenta, and *Ppp1r1b* in cyan (representative of 7 mice). **(d)** FISH for *Drd2* in green, *Grpr* in magenta, and *Drd1* in cyan (representative of 8 mice). **(e)** FISH for *Drd2* in green, *Grpr* in magenta, and *Penk* in cyan (representative of 8 mice). **(f-j)** Mean ± SEM percentage of *Grpr*+ cells in the DS of WT mice that are **(f)** *Chat*+ (n=7 mice), **(g)** *Ppp1r1b*+ (n=7 mice), **(h)** *Drd2*+ (n=12 mice), **(i)** *Drd1*+ (n=8 mice), or **(j)** *Penk*+ (n=8 mice). For all bar graphs, dots represent values for individual mice. Source data are provided as a Source Data file. Related to Figure 2.

Supplementary Figure 7

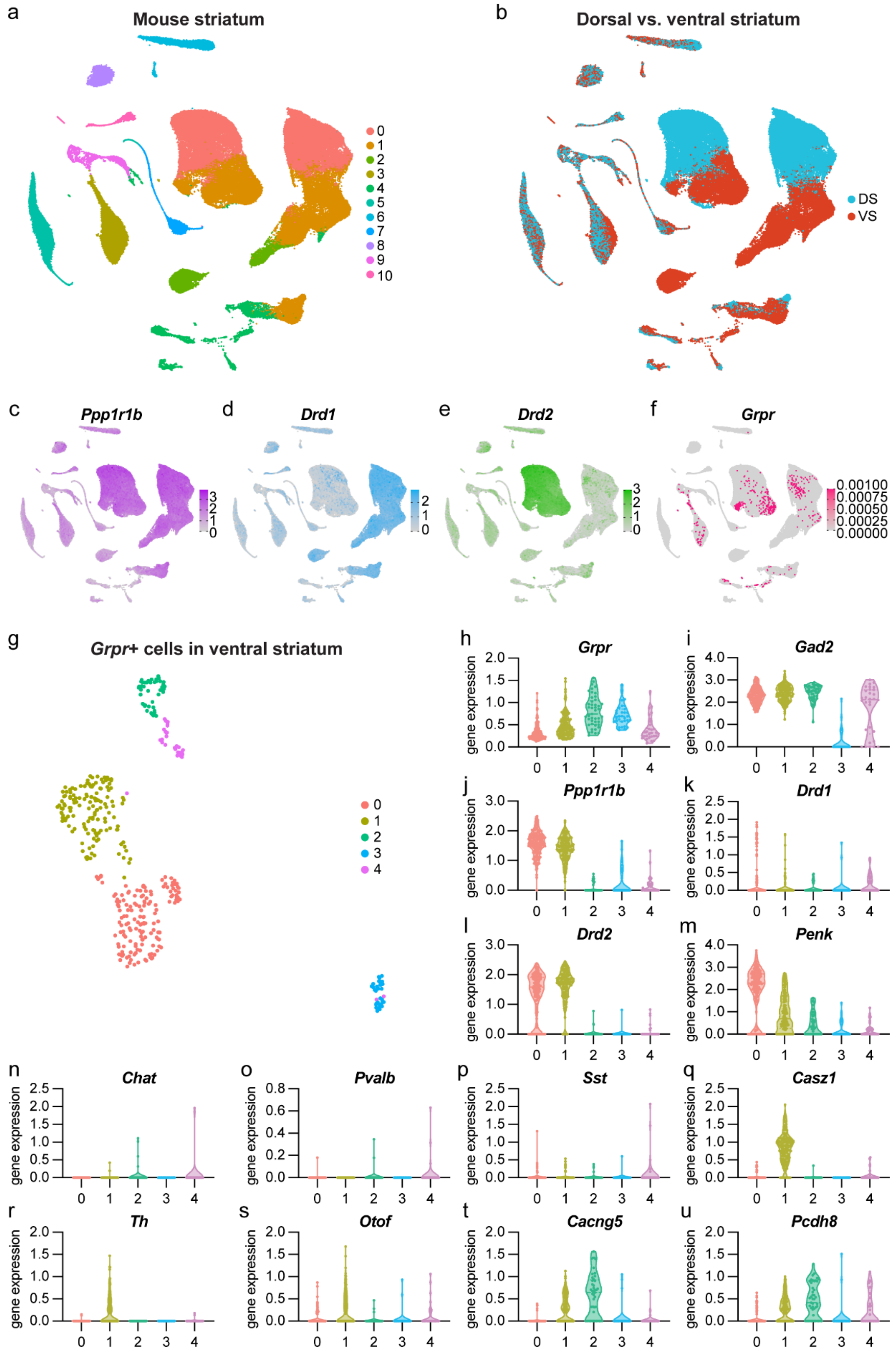

Supplementary Fig. 7: *Grpr* is expressed in multiple cell types in the mouse ventral striatum.

(a) UMAP of mouse striatum single-cell RNAseq data, colored by cluster. Data were re-clustered at a resolution of 0.1. Data and labels are from (Yao et al., 2023). Clusters map broadly to cell types as follows: 0: CNU-LGE GABA, 1: CNU-LGE GABA, 2: astrocytes, 3: CNU-LGE GABA, 4: glutamatergic neurons, 5: OPC-Oligodendrocytes, 6: Immune, 7: OPC-Oligodendrocytes, 8: vascular, 9: DG-IMN Glut/OB-IMN GABA, 10: vascular. **(b)** UMAP of mouse striatum single-cell data from **a**, colored by dissection region: DS=dorsal striatum (blue), VS=ventral striatum (ref). **(c-f)** Feature plots of **(c)** *Ppp1r1b*, **(d)** *Drd1*, **(e)** *Drd2*, and **(f)** *Grpr* expression the mouse striatum. For **f** the color scale was adjusted to highlight any cell expressing *Grpr* transcripts. **(g)** UMAP of *Grpr*-expressing cells in the VS. All cells expressing any *Grpr* were subsetted, re-processed, and re-clustered at a resolution of 0.4. **(h-u)** Violin plots showing the relative expression levels of **(h)** *Grpr*, **(i)** *Gad2*, **(j)** *Ppp1r1b*, **(k)** *Drd1*, **(l)** *Drd2*, **(m)** *Penk*, **(n)** *Chat*, **(o)** *Pvalb*, **(p)** *Sst*, **(q)** *Casz1*, **(r)** *Th*, **(s)** *Otof*, **(t)** *Cacng5*, and **(u)** *Pcdh8* across the clusters identified in the UMAP of *Grpr*-expressing cells in **g**. For panels **h-u**, each dot represents the expression level in a single cell. Source data are provided as a Source Data file. Related to Figure 2.

Supplementary Figure 8

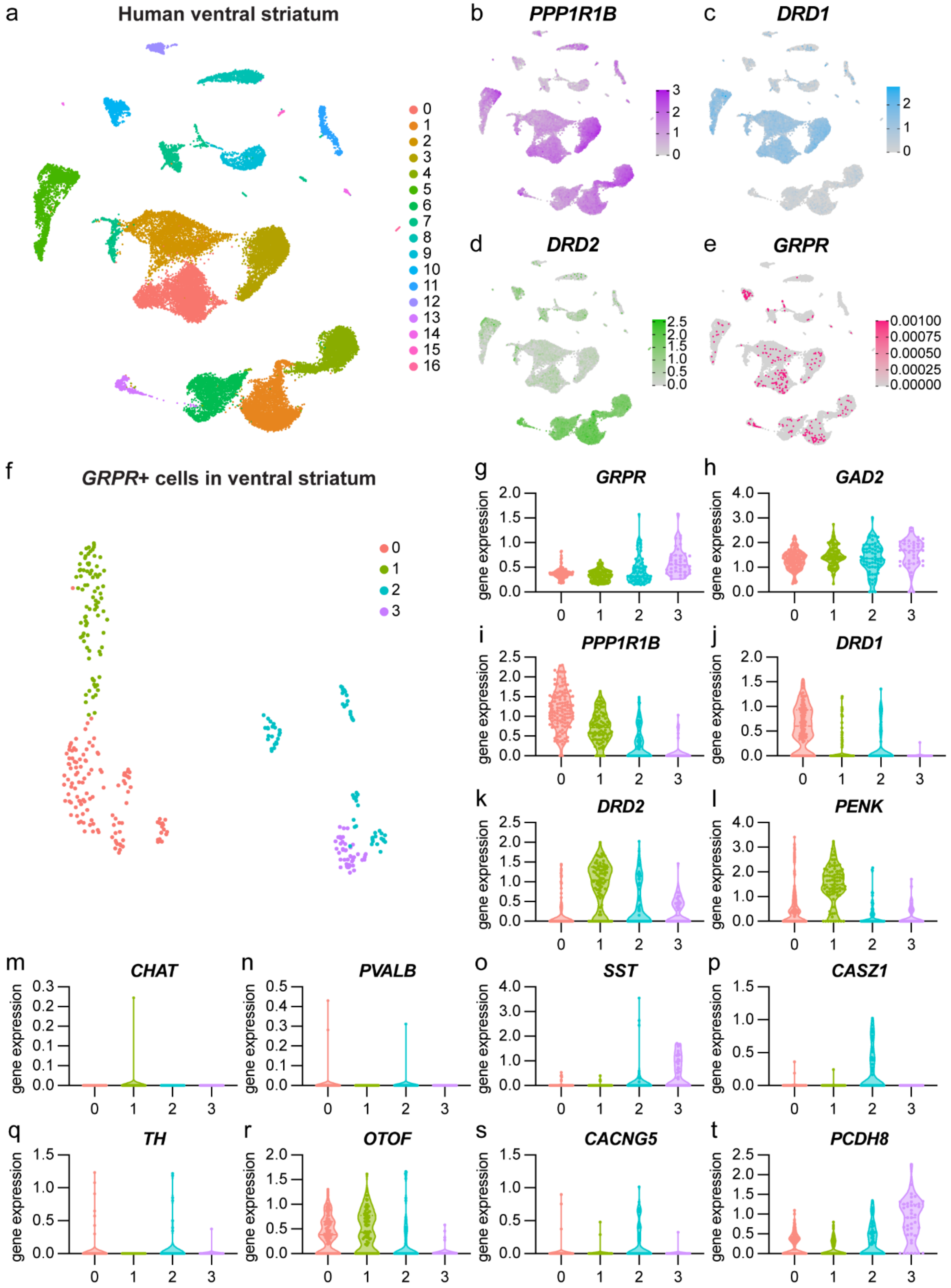

Supplementary Fig. 8: *GRPR* is expressed in multiple cell types in the human ventral striatum.

(a) UMAP of human ventral striatum single-cell RNAseq data, colored by cell type annotation. Data and labels are from (Siletti et al., 2023). Clusters map broadly to cell types as follows (terminology from Siletti et al.): 0,1,2,3,4,6,13 striatal projection neurons; 5 eccentric striatal projection neurons; 7,9,10,16 splatter; 8 oligodendrocytes; 11 astrocytes, 12 oligodendrocyte precursor cells; 14 microglia; 15 ependymal. **(b-e)** Feature plots of **(b)** *PPP1R1B*, **(c)** *DRD1*, **(d)** *DRD2*, and **(e)** *GRPR* expression in the human striatum. For **e** the color scale was adjusted to highlight any cell expressing *GRPR* transcripts. **(f)** UMAP of *GRPR*-expressing cells in the ventral striatum. All cells expressing any *GRPR* were subsetted, re-processed, and re-clustered at a resolution of 0.3. **(g-t)** Violin plots showing relative expression levels of **(g)** *GRPR*, **(h)** *GAD2*, **(i)** *PPP1R1B*, **(j)** *DRD1*, **(k)** *DRD2*, **(l)** *PENK*, **(m)** *CHAT*, **(n)** *PVALB*, **(o)** *SST*, **(p)** *CASZ1*, **(q)** *TH*, **(r)** *OTOF*, **(s)** *CACNG5*, and **(t)** *PCDH8* across the clusters identified in the UMAP of *GRPR*-expressing cells in **f**. For panels **g-t**, each dot represents the expression level in a single cell. Source data are provided as a Source Data file. Related to Figure 2.

Supplementary Figure 9

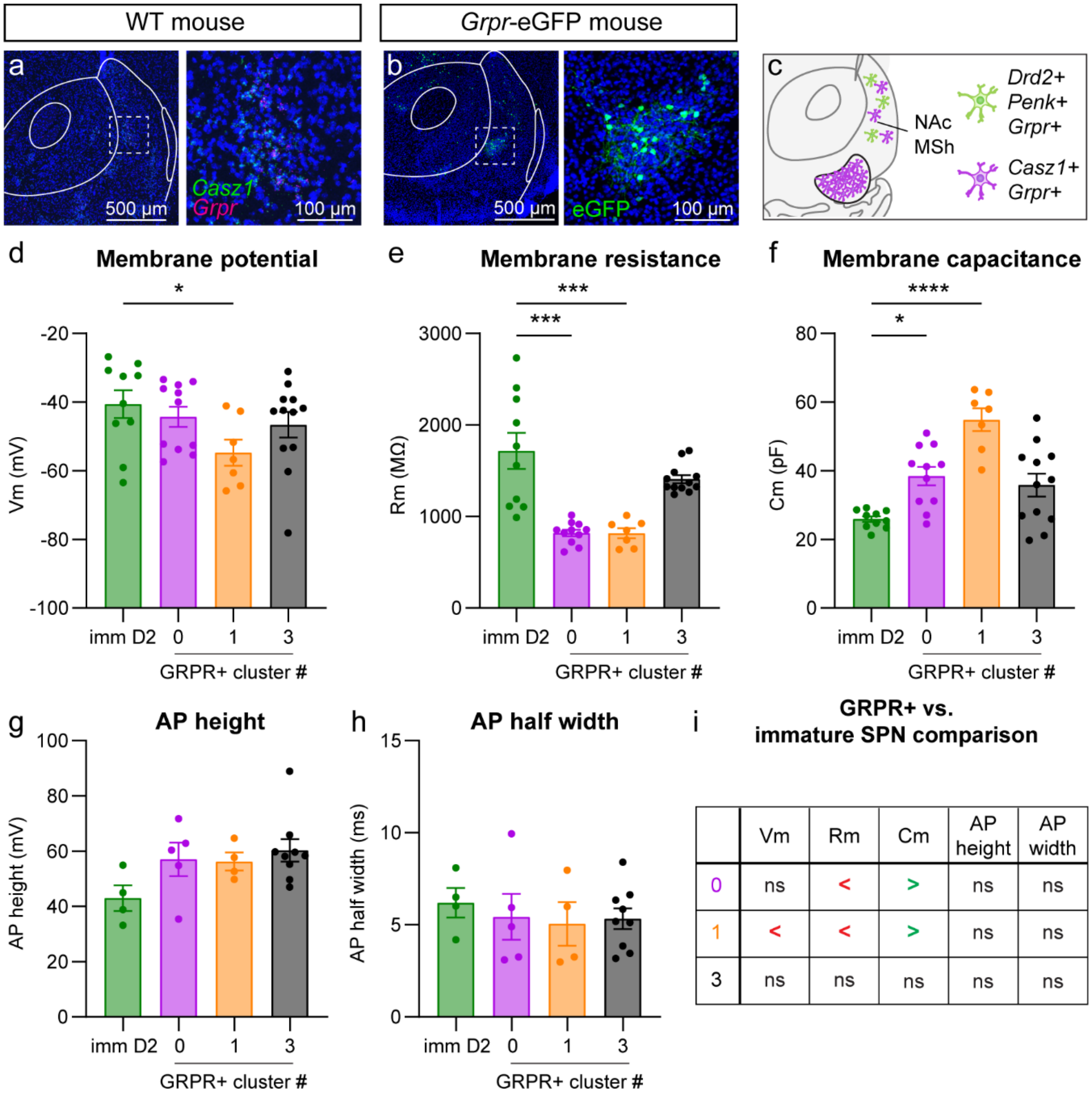

Supplementary Fig. 9: A subset of *Grpr*+ cells have similar properties to immature SPNs in the NAc MSh.

(a) Representative image of the NAc MSh (representative of 8 mice). FISH for *Casz1* in green and *Grpr* in magenta. Nuclei labeled with DAPI in blue. Right panel is a zoom-in from the dashed box on the left. **(b)** Representative image of the NAc MSh from a *Grpr*-eGFP mouse (representative of 6 mice). eGFP in green, DAPI labeled nuclei in blue. Right panel is a zoom-in from the dashed box on the left. **(c)** Summary schematic of the NAc MSh showing three different populations of *Grpr*+ cells based on their genetic markers and location: 1) sparsely distributed *Drd2+/Penk+/Grpr+* cells (green), 2) sparsely distributed *Casz1+/Grpr+* cells (purple), 3) clustered *Casz1+/Grpr+* cells (purple cells in the outlined ventral region). **(d)** Mean ± SEM resting membrane potential of NAc MSh neurons. P8-P12 D2-GFP+ (imm D2) cells in dark green, *Grpr*-eGFP+ cells in cluster 0 in purple, *Grpr*-eGFP+ cells in cluster 1 in orange, and *Grpr*-eGFP+ cells in cluster 3 in black. **(e)** Mean ± SEM membrane resistance. **(f)** Mean ± SEM membrane capacitance. **(g)** Mean ± SEM AP height. **(h)** Mean ± SEM AP half-width. **(i)** Summary table of **d**-**h**. Data for GRPR+ clustered cells in **d**-**h** are duplicated from Fig. 3 for comparison. For all bar graphs, dots represent values for individual cells. Source data are provided as a Source Data file. See the Supplementary Data file for sample sizes and statistics. Related to Figure 3.

Supplementary Figure 10

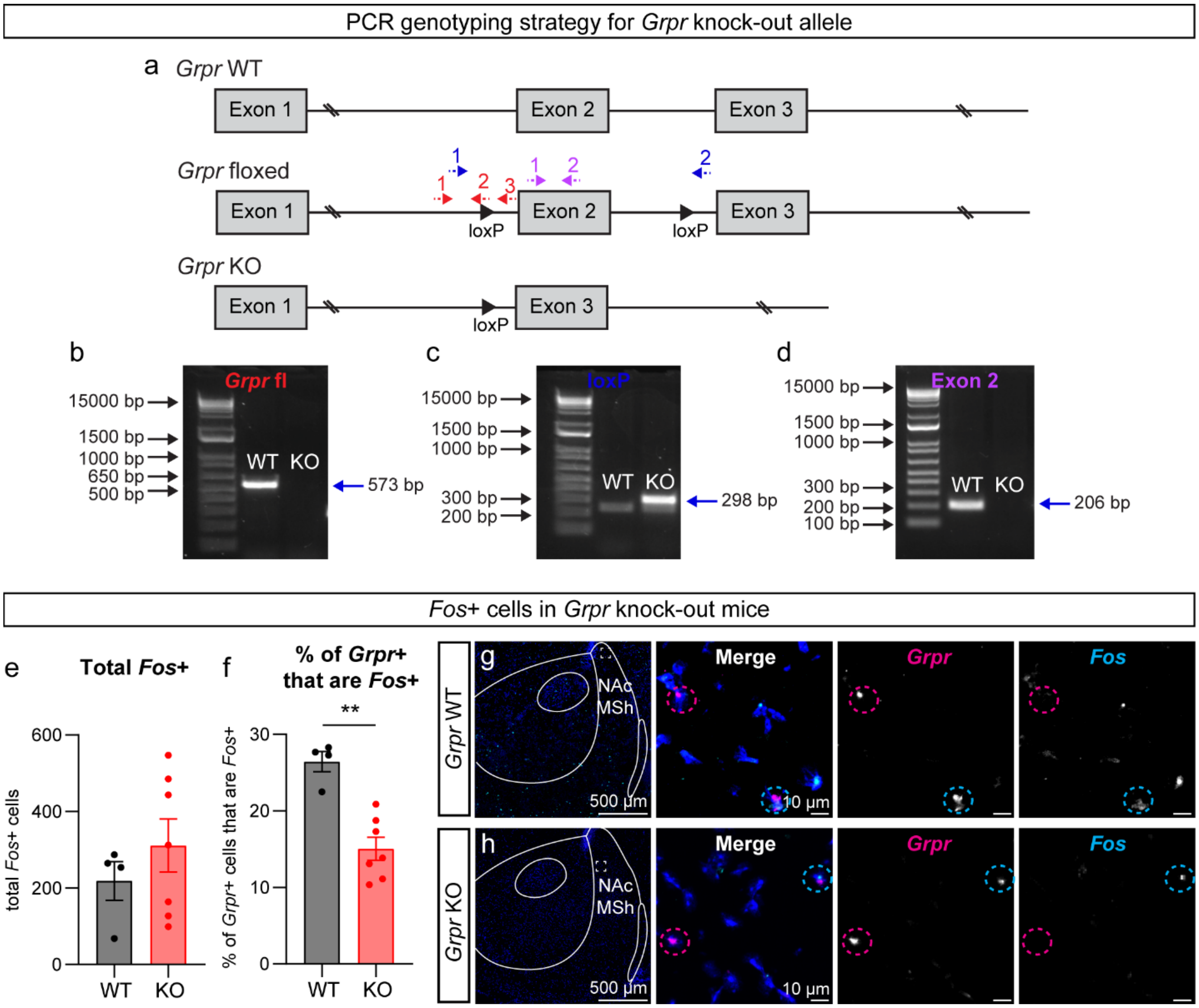

Supplementary Fig. 10: Mice lacking *Grpr* show reduced *Fos* expression in the NAc MSh.

Grpr floxed mice were bred with CMV-Cre mice to delete *Grpr*. *Grpr* is on the X chromosome and only male mice were used for this analysis (*Grpr*$^{fl/y}$). **(a)** Genotyping strategy for detection of the *Grpr* knock-out (KO), wild-type (WT), and floxed alleles. Three primers (red) were designed to detect the presence of the loxP site within the floxed allele (1-WT forward, 2-floxed reverse, and 3-WT reverse). Two primers (blue) were designed to detect the loxP scar in the KO allele (1-loxP forward and 2-loxP reverse). Two primers (purple) were designed to detect exon 2 (1-Exon 2 forward and 2-Exon 2 reverse). **(b)** PCR detecting the presence of the floxed or WT allele for *Grpr*. WT mice were expected to have a 573 base pair (bp) band and KO mice to have no band. **(c)** PCR detecting the loxP scar, indicating successful recombination. WT mice were not expected to have a band as the sequence is too large to be amplified. KO mice should have a band at 298 bp. **(d)** PCR for the presence of *Grpr* exon 2. WT mice were expected to have a band at 206 bp. KO mice should not have a band. **(e)** Mean ± SEM total number of *Fos*+ cells in the NAc MSh in WT (black) and *Grpr* KO (red) mice. (n=4 WT and n=7 KO mice, p=0.4121, two-tailed Mann-Whitney test). **(f)** Mean ± SEM percent *Grpr*+ cells in the NAc MSh that are *Fos*+ in *Grpr* WT (black) and *Grpr* KO (red) mice. (n is the same as panel **e**, **p=0.0061, two-tailed Mann-Whitney test). For **e** and **f**, dots represent values for individual mice. **(g-h)** Representative images of the NAc MSh from a *Grpr* WT mouse (**g**, representative of 4 mice) and a *Grpr* KO mouse (**h**, representative of 7 mice). FISH for *Grpr* in magenta and *Fos* in cyan. Dashed circles outline *Grpr*+ cells, magenta=*Grpr*+/*Fos*- and cyan=*Grpr*+/*Fos*+. Source data are provided as a Source Data file. Related to Figure 4.

Supplementary Figure 11

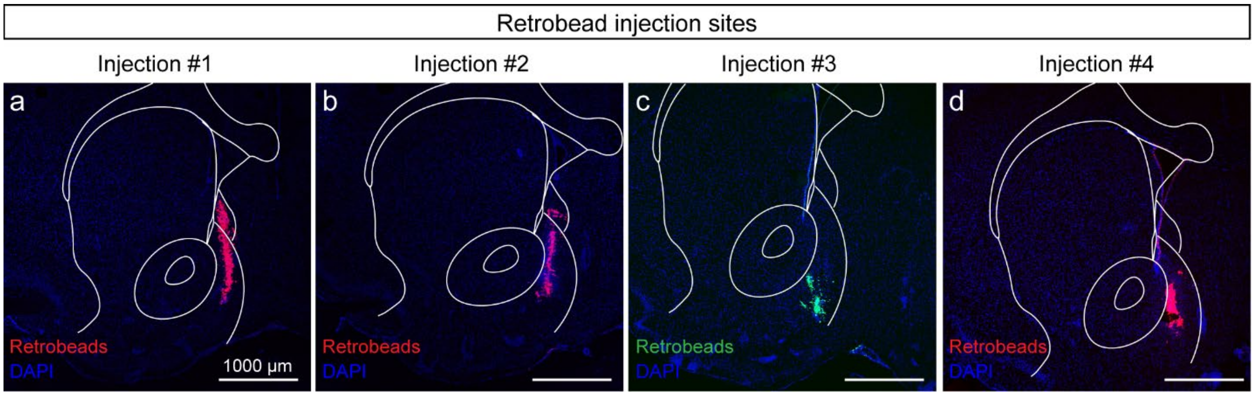

Supplementary Fig. 11: Images of retrobead injection sites in the NAc MSh.

(a-d) Images showing red or green fluorescent retrobead injection sites in the NAc MSh with DAPI staining in blue. Four independent injections were made across three mice. Related to Figure 5.

Supplementary Figure 12

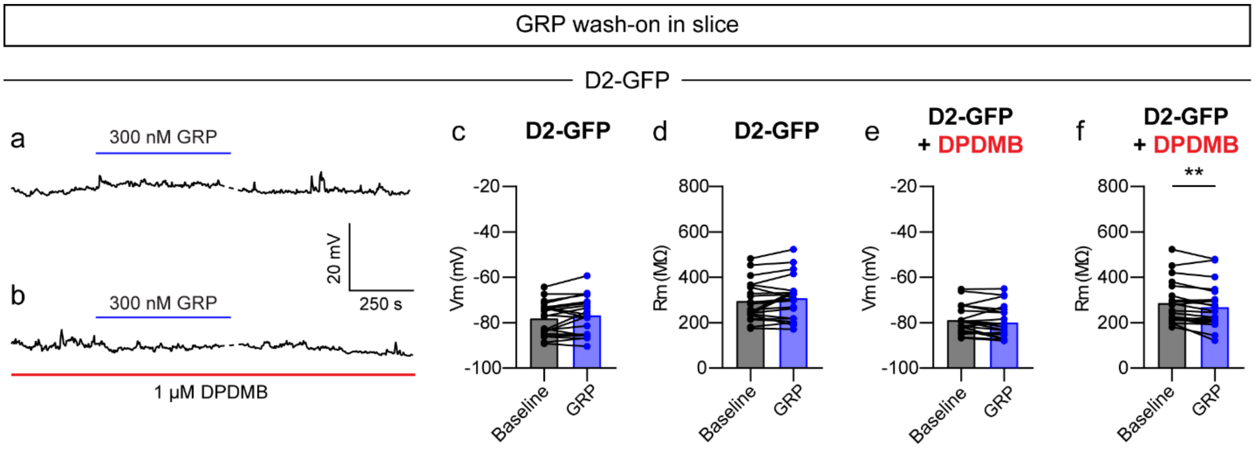

Supplementary Fig. 12: GRP does not increase the excitability of the general population of NAc MSh D2-expressing neurons.

(a) Representative trace showing the membrane potential over time of a NAc MSh D2-GFP+ cell (representative of 20 neurons). Blue bar represents ten-minute wash-on of 300 nM GRP. **(b)** Representative trace showing the membrane potential over time of a NAc MSh D2-GFP+ cell (representative of 22 neurons). 300 nM GRP (blue bar) was washed-on in the presence of 1 µM DPDMB (red bar, entirety of recording). **(c)** Membrane potential of D2-GFP+ NAc MSh neurons at baseline (black) and after 300 nM GRP wash-on (blue); (n=20 cells from 20 mice; p=0.1054, two-tailed Wilcoxon test). **(d)** Membrane resistance of D2-GFP+ NAc MSh neurons at baseline and after GRP wash-on; (n=20 cells from 20 mice; p=0.1429, two-tailed Wilcoxon test). **(e)** Membrane potential of D2-GFP+ NAc MSh neurons at baseline and after GRP wash-on in the presence of DPDMB; (n=22 cells from 17 mice; p=0.0501, two-tailed Wilcoxon test). **(f)** Membrane resistance of D2-GFP+ NAc MSh neurons at baseline and after GRP wash-on in the presence of DPDMB; (n=22 cells from 17 mice; **p=0.0025, two-tailed Wilcoxon test). For all bar graphs, bars represent the mean and dots represent values for individual cells. Source data are provided as a Source Data file. Related to Figure 6.

Supplementary Figure 13

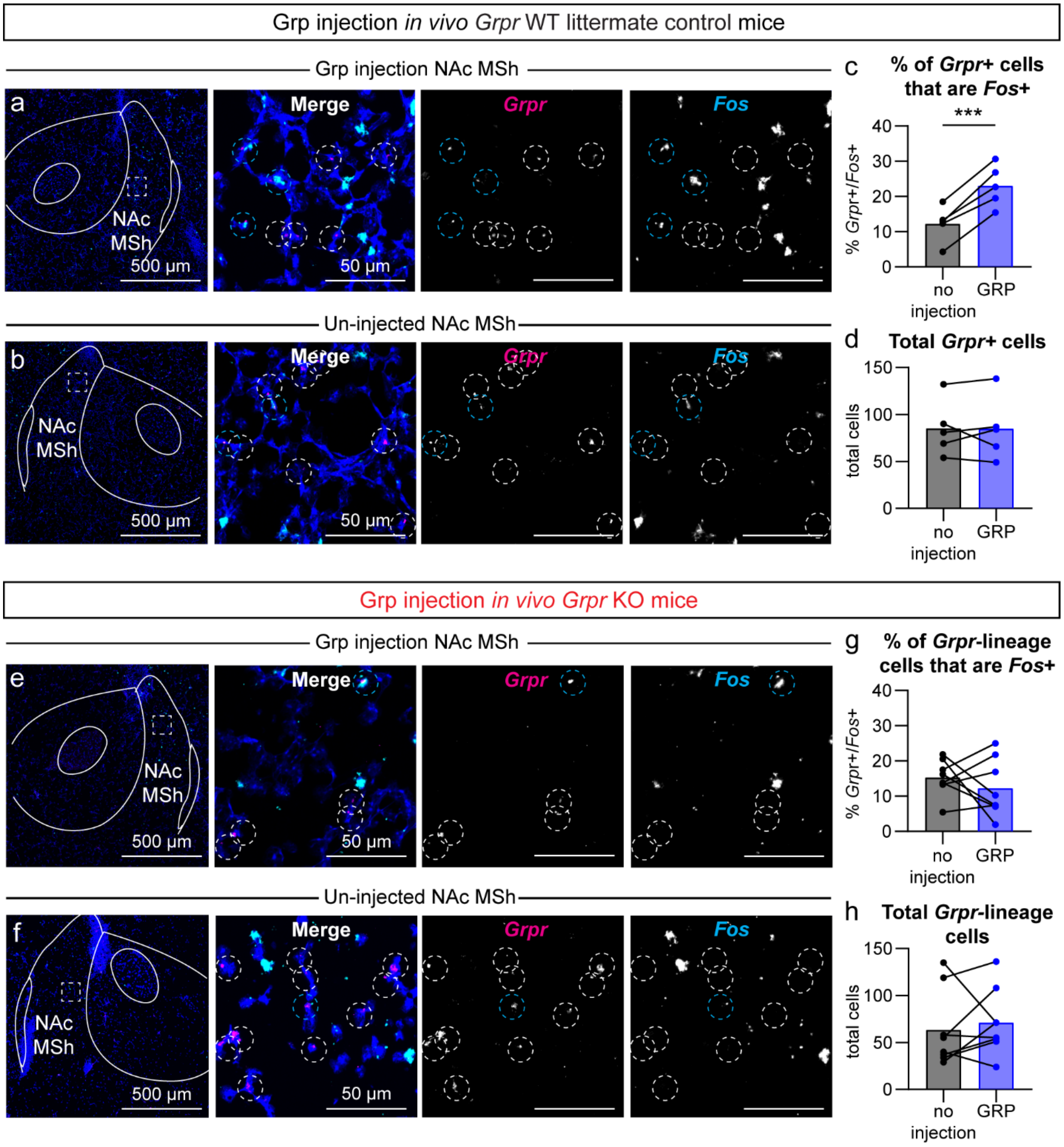

Supplementary Fig. 13: *Grpr* KO mice do not have increased *Fos* expression in *Grpr*-lineage cells in response to GRP injection *in vivo*.

(a-b) Representative images of the NAc MSh from *Grpr* wild-type (WT) mice from the **(a)** injected hemisphere (3 μM GRP) and **(b)** un-injected hemisphere (representative of 5 mice). FISH for *Grpr* in magenta and *Fos* in cyan. Nuclei are labeled with DAPI in blue. Dashed circles outline *Grpr*+ cells, white=*Grpr*+/*Fos*-, cyan=*Grpr*+/*Fos*+. **(c)** Percentage of *Grp*r+ cells in the NAc MSh that were *Fos*+ in the un-injected vs GRP-injected hemisphere of *Grpr* WT mice. (n=5 mice, ***p=0.0007, two-tailed paired t-test). **(d)** Total number of *Grpr*+ cells in the NAc MSh in the un-injected vs GRP-injected hemisphere of *Grpr* WT mice (n=5 mice, p=0.9552, two-tailed paired t-test). **(e-f)** Representative images of the NAc MSh from *Grpr* knock-out (KO) mice from the **(e)** GRP injected hemisphere and **(f)** un-injected hemisphere (representative of 8 mice). **(g)** Percentage of *Grp*r-lineage cells in the NAc MSh that were *Fos*+ in the un-injected vs GRP-injected hemisphere of *Grpr* KO mice (n=8 mice, p=0.4000, two-tailed paired t-test). **(h)** Total number of *Grpr*-lineage cells in the NAc MSh in the un-injected vs GRP-injected hemisphere of *Grpr* KO mice (n=8 mice, p=0.5606, two-tailed paired t-test). For panels **c-d** and **g-h**, data were summed across two sections per mouse. For all graphs, bars represent the mean and dots represent values for individual mice. Source data are provided as a Source Data file. Related to Figure 6.

Supplementary Figure 14

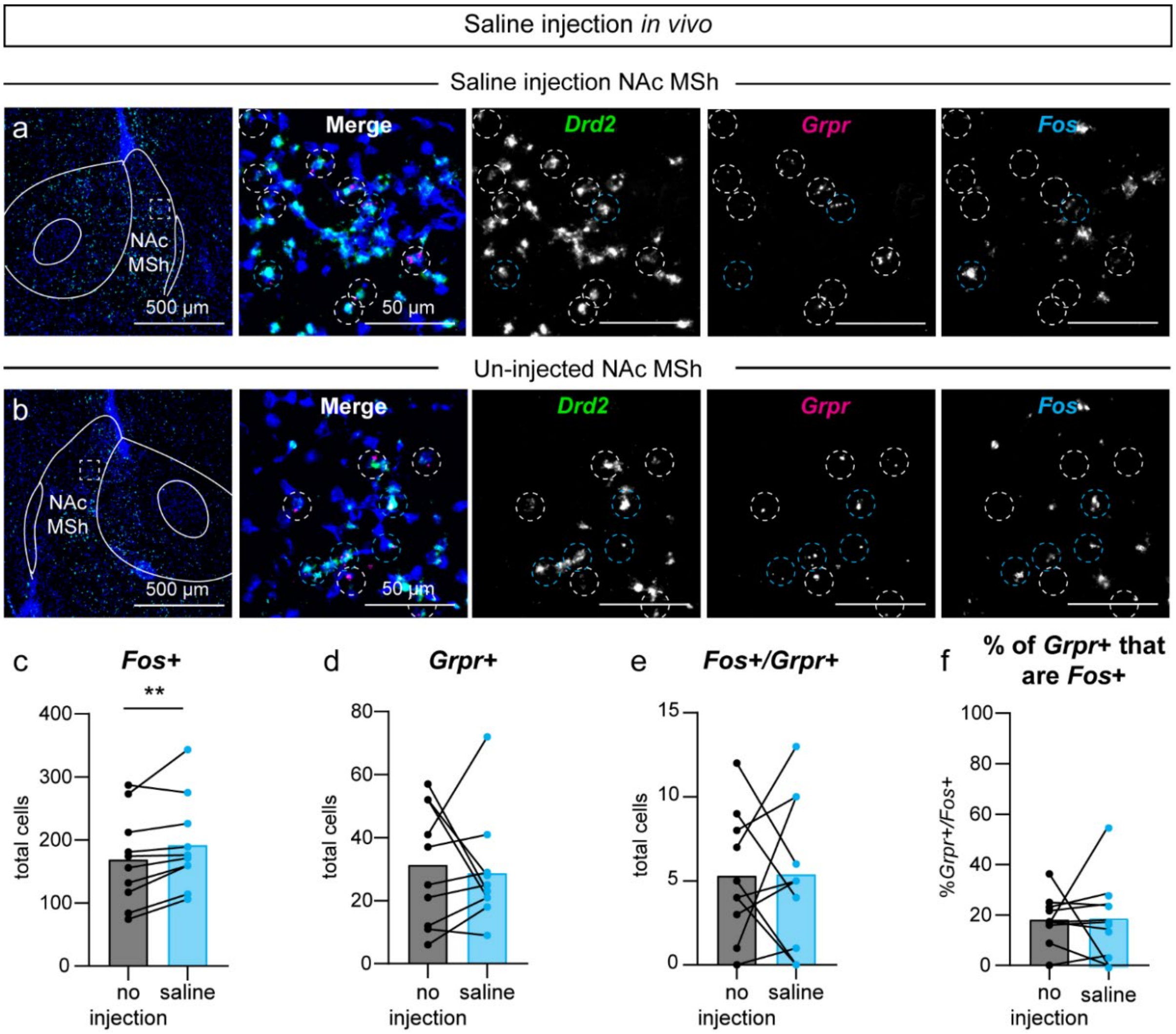

Supplementary Fig. 14: Saline injection into the NAc MSh does not induce *Fos* in *Grpr*-expressing cells.

(a-b) Representative images of the NAc MSh from the **(a)** saline-injected hemisphere and **(b)** un-injected hemisphere (representative of 10 mice). FISH for *Drd2* in green, *Grpr* in magenta, and *Fos* in cyan. Nuclei are labeled with DAPI in blue. Dashed circles outline *Grpr*+ cells, white=*Grpr*+/*Fos*-, cyan=*Grpr*+/*Fos*+. **(c)** Total number of cells in the NAc MSh that were *Fos*+ in the un-injected (black) vs saline-injected hemisphere (light blue) (**p=0.0098, two-tailed Wilcoxon test). **(d)** Total number of cells in the NAc MSh that were *Grpr*+ in the un-injected vs saline-injected hemisphere (p=0.9414, two-tailed Wilcoxon test). **(e)** Total number of *Grpr*+ cells in the NAc MSh that were *Fos*+ in the un-injected vs saline-injected hemisphere (p=0.9570, two-tailed Wilcoxon test). **(f)** Percentage of *Grp*r+ cells in the NAc MSh that were *Fos*+ in the un-injected vs saline-injected hemisphere (p=0.7695, two-tailed Wilcoxon test). For panels **c-f** data were summed from

two coronal sections per mouse (n=10 mice). For all graphs, bars represent the mean and dots represent values for individual mice. Source data are provided as a Source Data file. Related to Figure 6.

Supplementary Figure 15

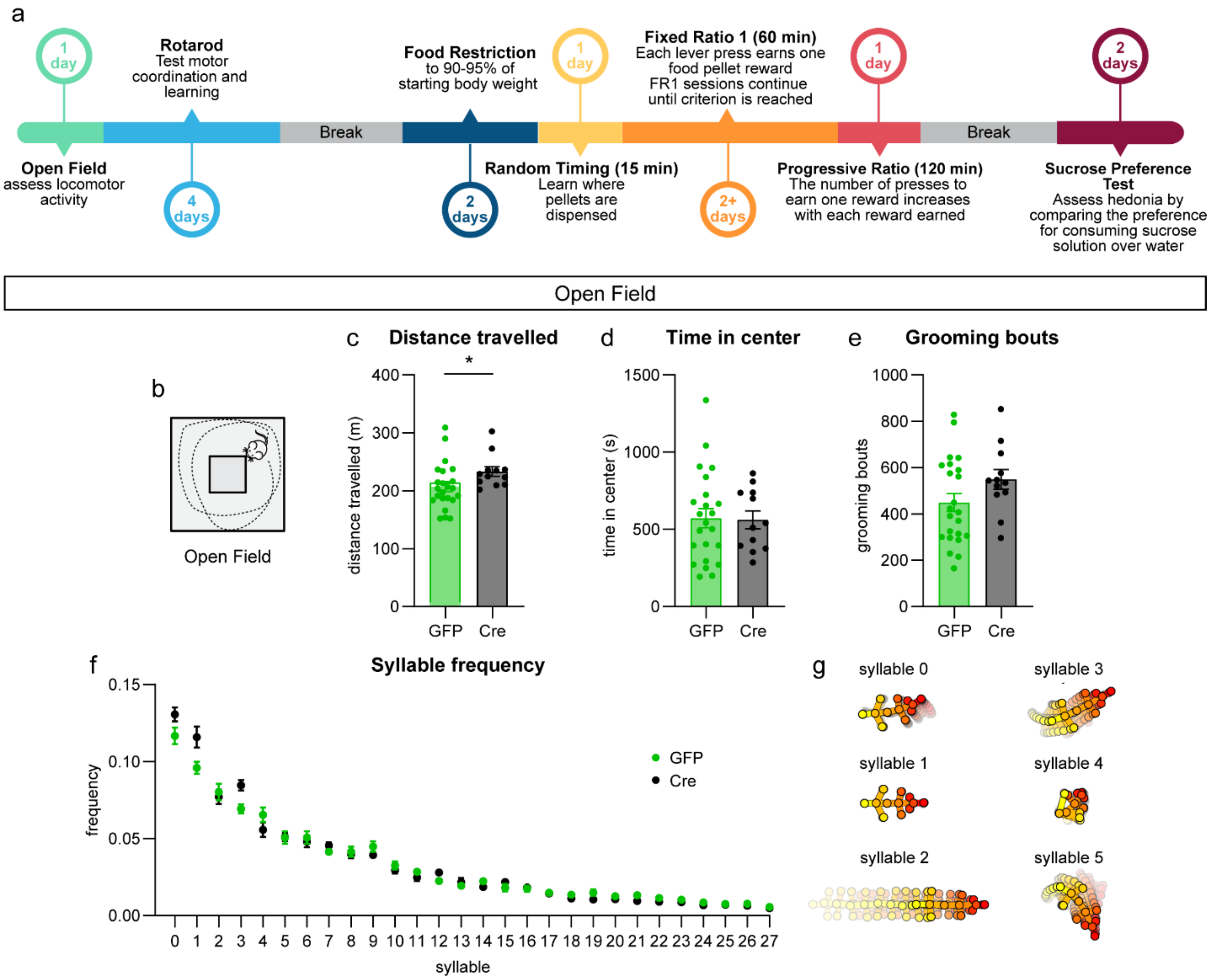

Supplementary Fig. 15: Mice lacking *Grpr* in the NAc MSh have increased locomotor activity but no difference in behavioral syllable usage in the open field.

(a) Timeline of behavioral assays in *Grpr* conditional knock-out (cKO) and littermate control mice. **(b)** Schematic of the open field test. **(c)** Mean ± SEM total distance travelled in a one-hour session for control (GFP-injected, green) and *Grpr* cKO (Cre-injected, black) mice (two-tailed Mann-Whitney test, *p=0.0234; n=23 GFP and 12 Cre mice for all panels). **(d)** Mean ± SEM total time spent in the center of the open field during the one-hour session. Center was defined as a 20 cm x 20 cm square in the middle of the arena (two-tailed Mann-Whiney test, p=0.8506). **(e)** Mean ± SEM number of grooming bouts during the one-hour session (two-tailed Mann-Whitney test, p=0.1723). **(f)** Mean ± SEM frequency of syllable usage for control (GFP-injected, green) and *Grpr* cKO (Cre-injected, black) mice. Syllable descriptions: 0-left turn, 1-static, 2-forward, 3-left turn 2, 4-grooming, 5-right turn, 6-forward 2, 7-left turn 3, 8-forward left, 9-right turn 2, 10-forward 3, 11-grooming 2, 12-grooming 3, 13-grooming 4, 14-backward right, 15-backward left, 16-forward 4, 17-left turn 4, 18-grooming 5, 19-forward 5, 20-forward 6, 21-forward 7, 22-backward, 23-forward 8, 24-forward right, 25-right turn 3, 26-grooming 6, 27-left turn 5. Kruskal-Wallis with Dunn's correction for multiple comparisons, 0: p=0.1724,1: p=0.1540, 2: p=0.5629, 3: p=0.1498, 4: p=0.2520, 5: p=0.8470, 6: p=0.6170, 7: p=0.2589, 8: p=0.7552, 9: p=0.4081, 10: p=0.5152, 11: p=0.2021, 12: p=0.1498, 13: p=0.8276, 14: p=0.1540, 15: p=0.2021, 16: p=0.7552, 17: p=0.6745, 18: p=0.1782, 19: p=0.1540, 20: p=0.5186, 21: p=0.2021, 22: p=0.2180, 23: p=0.5214, 24: p=0.1540, 25: p=0.5629, 26: p=0.4716, 27: p=0.2180. **(g)** Average pose trajectories for the top six Keypoint-MoSeq syllables. Each trajectory includes ten poses, starting 165 ms before and ending 500 ms after syllable onset. For all bar graphs, dots represent values for individual mice. Source data are provided as a Source Data file. Related to Figure 7.

Supplementary Figure 16

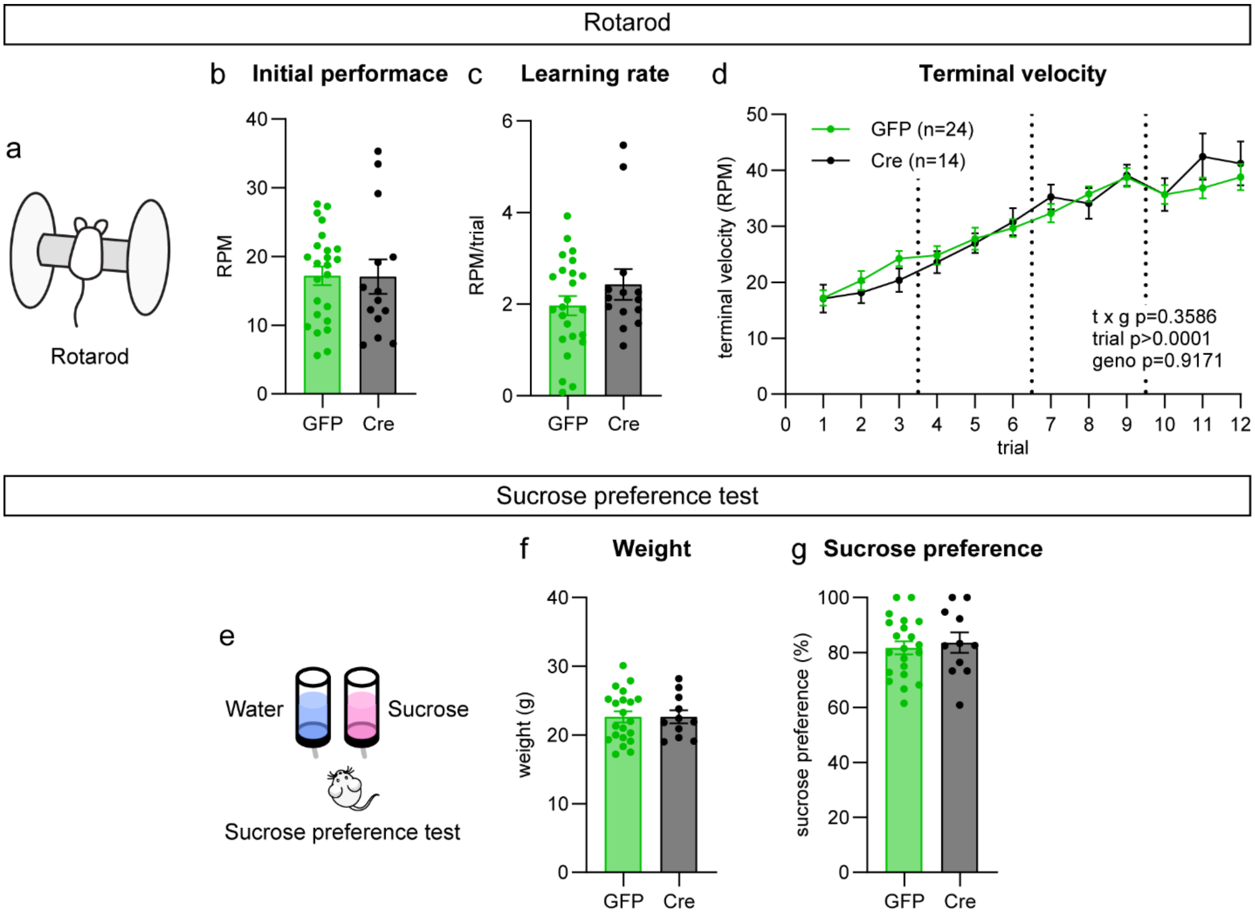

Supplementary Fig. 16: Mice lacking *Grpr* in the NAc MSh show no differences in the rotarod or sucrose preference tests.

(a) Schematic of the accelerating rotarod. **(b)** Mean ± SEM performance on trial 1 of the rotarod in control (GFP-injected, green) vs *Grpr* cKO (Cre-injected, black) mice (two-tailed Mann-Whitney test, p=0.6700; n=24 GFP and 14 Cre mice). **(c)** Mean ± SEM of learning rate, measured as the slope of the linear regression of the performance per mouse across all trials (two-tailed Mann-Whitney test, p=0.5201; n is the same as in panel **b**) **(d)** Mean ± SEM rotarod performance for GFP-injected and Cre-injected mice across all trials, measured as terminal velocity. Two-way repeated measures ANOVA p-values are shown in the graph. (n is the same as for panel **b**). **(e)** Schematic of sucrose preference test. **(f)** Mean ± SEM weight of GFP-injected and Cre-injected mice just prior to the sucrose preference test (two-tailed Mann-Whitney test, p=0.9300; n=21 GFP and 11 Cre mice). **(g)** Mean ± SEM sucrose preference measured as the percentage of total liquid consumed that was 5% sucrose (Welch's two-tailed t-test, p=0.6785; n is the same as for panel **f**). For all bar graphs, dots represent values for individual mice. Source Data are provided as a Source Data file. Related to Figure 7.

Supplementary Figure 17

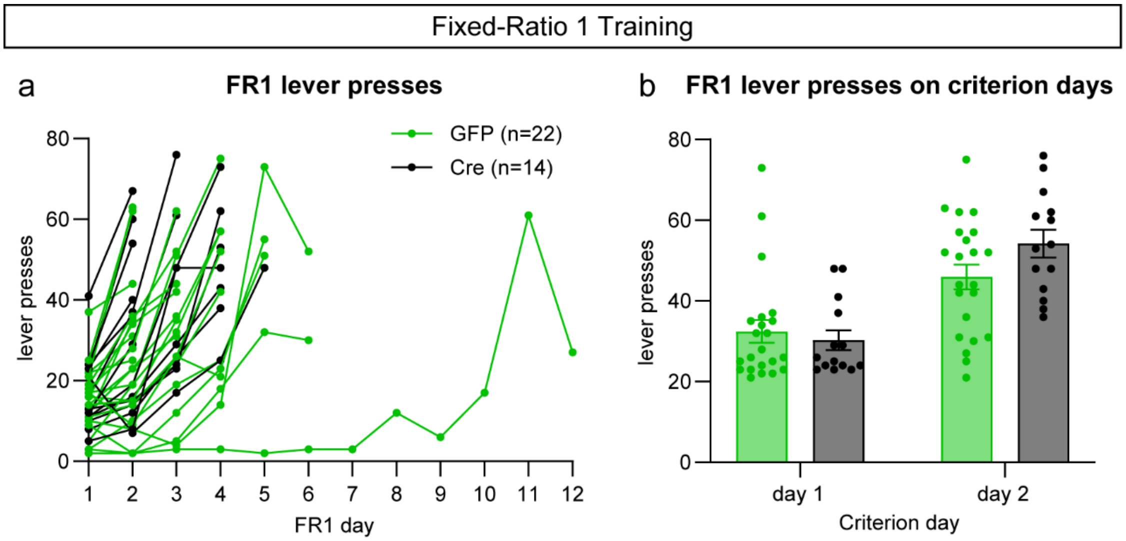

Supplementary Fig. 17: Mice lacking *Grpr* in the NAc MSh have normal performance in fixed ratio training sessions.

(a) Total number of lever presses on each day of the fixed-ratio 1 (FR1) training for individual mice. Control (GFP-injected) mice are shown in green and *Grpr* conditional knock-out (cKO, Cre-injected) mice are shown in black. n=22 GFP and 14 Cre mice for all panels. **(b)** Mean ± SEM total lever presses on the final two days of FR1 training in control (GFP-injected, green) and *Grpr* cKO (Cre-injected, black) mice (Two-way repeated measures ANOVA: day x genotype p=0.0671, day ****p<0.0001, genotype p=0.3845; Holm-Sidak's multiple comparisons test, day 1: p=0.8596, day 2: p=0.1278). Dots represent values for individual mice. Source data are provided as a Source Data file. Related to Figure 7.
