# Supplementary material for: Gastrin-releasing peptide signaling in the nucleus accumbens medial shell regulates neuronal excitability and motivation
Source: Nat Commun. 2025 Oct 21;16:9314. doi: 10.1038/s41467-025-64373-3 (PMC12540653; doi:10.1038/s41467-025-64373-3)
Supplement: Supplementary file 2 — Description of Additional Supplementary Files [file 41467_2025_64373_MOESM2_ESM.pdf]

**Description of Additional Supplementary Files**

**Supplementary Data:**

Sample sizes and statistical information for Figures 1, 3, 4 and 6 and Supplementary Fig. 9.
