# Supplementary material for: Gastrin-releasing peptide signaling in the nucleus accumbens medial shell regulates neuronal excitability and motivation
Source: Nat Commun. 2025 Oct 21;16:9314. doi: 10.1038/s41467-025-64373-3 (PMC12540653; doi:10.1038/s41467-025-64373-3)
Supplement: Supplementary file 4 — Reporting Summary [file 41467_2025_64373_MOESM4_ESM.pdf]

nature portfolio

Corresponding author(s): Helen Bateup

Last updated by author(s): June 21, 2025

Reporting Summary

Nature Portfolio wishes to improve the reproducibility of the work that we publish. This form provides structure for consistency and transparency in reporting. For further information on Nature Portfolio policies, see our Editorial Policies and the Editorial Policy Checklist.

Statistics

For all statistical analyses, confirm that the following items are present in the figure legend, table legend, main text, or Methods section.

n/a	Confirmed	
☐	☒	The exact sample size (*n*) for each experimental group/condition, given as a discrete number and unit of measurement
☐	☒	A statement on whether measurements were taken from distinct samples or whether the same sample was measured repeatedly
☐	☒	The statistical test(s) used AND whether they are one- or two-sided<br>*Only common tests should be described solely by name; describe more complex techniques in the Methods section.*
☒	☐	A description of all covariates tested
☐	☒	A description of any assumptions or corrections, such as tests of normality and adjustment for multiple comparisons
☐	☒	A full description of the statistical parameters including central tendency (e.g. means) or other basic estimates (e.g. regression coefficient) AND variation (e.g. standard deviation) or associated estimates of uncertainty (e.g. confidence intervals)
☐	☒	For null hypothesis testing, the test statistic (e.g. *F*, *t*, *r*) with confidence intervals, effect sizes, degrees of freedom and *P* value noted<br>*Give P values as exact values whenever suitable.*
☒	☐	For Bayesian analysis, information on the choice of priors and Markov chain Monte Carlo settings
☒	☐	For hierarchical and complex designs, identification of the appropriate level for tests and full reporting of outcomes
☐	☒	Estimates of effect sizes (e.g. Cohen's *d*, Pearson's *r*), indicating how they were calculated

Our web collection on statistics for biologists contains articles on many of the points above.

Software and code

Policy information about availability of computer code

Data collection

Confocal images were collected with Olympus FluoView software. Electrophysiology data were acquired with ScanImage (https://github.com/bernardosabatini/SabalabAcq). Operant conditioning data was acquired with Med Associates software. Open field data were collected using a FLIR Grasshopper 3 monochrome camera. Rotarod data was collected using Ugo Basille software.

Data analysis

Confocal image analysis was done in Image J (versions 1.53-1.54). Spine density analysis was done in Imaris (version 9.3.1). Published single cell RNA sequencing datasets were analyzed using Seurat V5. Open field data were analyzed with DeepLabCut (version 2.3.4) and Keypoint-MoSeq (version 0.4.4). Electrophysiology data were analyzed with Igor Pro (Wavemetrcs). Electrophysiology data were further analyzed using the UMAP-learn and Scikit-leran Python packages. Data were pre-processed using the StandardScaler function, and UMAP embeddings were calculated using the following parameters: (n_neighbors=10, min_dist=0, metric='euclidean'). Clustering was performed on the scaled dataset using the K-means approach with an n_clusters value of 4.

See https://github.com/BateupLab/Aisenberg2025 for custom analysis code used in this study (DOI: 10.5281/zenodo.15725043).

For manuscripts utilizing custom algorithms or software that are central to the research but not yet described in published literature, software must be made available to editors and reviewers. We strongly encourage code deposition in a community repository (e.g. GitHub). See the Nature Portfolio guidelines for submitting code & software for further information.

Data

Policy information about availability of data

All manuscripts must include a data availability statement. This statement should provide the following information, where applicable:

- Accession codes, unique identifiers, or web links for publicly available datasets
- A description of any restrictions on data availability
- For clinical datasets or third party data, please ensure that the statement adheres to our policy

Source data are provided with this paper in the Source Data file. The mouse striatum single-cell RNA sequencing data are available in from the Allen Brain Cell Atlas under the accession code "WMB-10Xv3" (https://alleninstitute.github.io/abc_atlas_access/descriptions/WMB-10Xv3.html). The human nucleus accumbens single-cell sequencing data are available from the Human Brain Cell Atlas v1.0 at the CELLxGENE database under the name "Dissection: Basal nuclei (BN) - Nucleus Accumbens - NAC" (https://cellxgene.cziscience.com/collections/283d65eb-dd53-496d-adb7-7570c7caa443).

Research involving human participants, their data, or biological material

Policy information about studies with human participants or human data. See also policy information about sex, gender (identity/presentation), and sexual orientation and race, ethnicity and racism.

Reporting on sex and gender	*Use the terms sex (biological attribute) and gender (shaped by social and cultural circumstances) carefully in order to avoid confusing both terms. Indicate if findings apply to only one sex or gender; describe whether sex and gender were considered in study design; whether sex and/or gender was determined based on self-reporting or assigned and methods used. Provide in the source data disaggregated sex and gender data, where this information has been collected, and if consent has been obtained for sharing of individual-level data; provide overall numbers in this Reporting Summary. Please state if this information has not been collected. Report sex- and gender-based analyses where performed, justify reasons for lack of sex- and gender-based analysis.*
Reporting on race, ethnicity, or other socially relevant groupings	*Please specify the socially constructed or socially relevant categorization variable(s) used in your manuscript and explain why they were used. Please note that such variables should not be used as proxies for other socially constructed/relevant variables (for example, race or ethnicity should not be used as a proxy for socioeconomic status). Provide clear definitions of the relevant terms used, how they were provided (by the participants/respondents, the researchers, or third parties), and the method(s) used to classify people into the different categories (e.g. self-report, census or administrative data, social media data, etc.) Please provide details about how you controlled for confounding variables in your analyses.*
Population characteristics	*Describe the covariate-relevant population characteristics of the human research participants (e.g. age, genotypic information, past and current diagnosis and treatment categories). If you filled out the behavioural & social sciences study design questions and have nothing to add here, write "See above."*
Recruitment	*Describe how participants were recruited. Outline any potential self-selection bias or other biases that may be present and how these are likely to impact results.*
Ethics oversight	*Identify the organization(s) that approved the study protocol.*

Note that full information on the approval of the study protocol must also be provided in the manuscript.

Field-specific reporting

Please select the one below that is the best fit for your research. If you are not sure, read the appropriate sections before making your selection.

☒ Life sciences ☐ Behavioural & social sciences ☐ Ecological, evolutionary & environmental sciences

For a reference copy of the document with all sections, see nature.com/documents/nr-reporting-summary-flat.pdf

Life sciences study design

All studies must disclose on these points even when the disclosure is negative.

Sample size	No sample-size calculations were performed prior to the experiments. The sample sizes for experiments were determined to be adequate based on prior literature and the magnitude and consistency of measurable differences between groups. Electrophysiology data, FISH data, and GRP injections (PMID: 34610277 and PMID: 31103358). Mouse behavior (PMID: 29540712)
Data exclusions	For behavioral experiments, all Cre-injected animals were validated for injection targeting accuracy. Any animal for which the virus was not expressed in both hemispheres of the NAc MSh with minimal spread was excluded. For retrograde tracing experiments, only animals in which the injection was restricted to the NAc MSh were included. For electrophysiology experiments, cells were excluded if the series resistance increased above 30 MOhms during the recording.
Replication	All samples within each experiment are biological replicates (i.e. independent cells or mice). Datasets were collected once and all data

Replication	collected for each experiment were included in the analysis except for the exclusions listed above. Independent replication experiments were not carried out. For imaging experiments, similar expression patterns were observed across all mice examined.
Randomization	For electrophysiology recordings, cells and slices were randomly assigned to receive GRP or GRP + DPDMB. For the GRP in vivo injection experiments, mice were randomly assigned to receive either GRP or saline injection. For the conditional knock-out mouse experiments, Grprfl/fl or Grprfl/y mice were randomly assigned to be injected with AAV-GFP or AAV-Cre.
Blinding	Analysis of the in situ hybridization (FISH) images for Figure 2 and Supplementary Fig. 6 was done blind to the identity of the probes. The FISH analysis for WT and global KO mice was done blind to genotype (Supplementary Fig. 10). The spine analysis was done blind to cell type (Supplementary Fig. 2). Data collection for the behavior experiments was performed blind to genotype (Fig. 7, Supplementary Figs. 15-17). Electrophysiology experiments were not blinded due to the need to identify cell types by fluorophore expression.

Reporting for specific materials, systems and methods

We require information from authors about some types of materials, experimental systems and methods used in many studies. Here, indicate whether each material, system or method listed is relevant to your study. If you are not sure if a list item applies to your research, read the appropriate section before selecting a response.

Materials & experimental systems

n/a	Involved in the study
☐	☒ Antibodies
☒	☐ Eukaryotic cell lines
☒	☐ Palaeontology and archaeology
☐	☒ Animals and other organisms
☒	☐ Clinical data
☒	☐ Dual use research of concern
☒	☐ Plants

Methods

n/a	Involved in the study
☒	☐ ChIP-seq
☒	☐ Flow cytometry
☒	☐ MRI-based neuroimaging

Antibodies

Antibodies used	Primary antibodies:<br>RFP (1:500, rabbit, Rockland/VWR: RL600-401-379)<br>GFP (1:500 (spine imaging) or 1:5000 (IHC), chicken, Abcam: 13970)<br>Secondary antibodies:<br>Alexa Fluor 488 goat anti-chicken secondary antibody (1:500, ThermoFisher A-11039)<br>Alexa Fluor 546 goat anti-rabbit secondary antibody (1:500, ThermoFisher: A-11035)
Validation	All antibodies used for this study are commercially available and were validated by the manufacturer for the species and assays they were used for in this study.<br><br>RFP primary antibody validation: https://www.rockland.com/categories/primary-antibodies/rfp-antibody-pre-adsorbed-600-401-379/?srsltid=AfmBOopEMpZPC_WnWda592DywB4-pUA_rKRKbJrWb6_0WTC9t90psFRB<br><br>GFP primary antibody validation: https://www.abcam.com/en-us/products/primary-antibodies/gfp-antibody-ab13970?srsltid=AfmBOoqaud_RyJXhRiGkJzQ4ZcSVU5Rsi1M-Bs1FOMiRo5f3-_MTug73

Animals and other research organisms

Policy information about studies involving animals; ARRIVE guidelines recommended for reporting animal research, and Sex and Gender in Research

Laboratory animals	Animal experiments were performed in accordance with protocols approved by the University of California, Berkeley, Institutional Animal Care and Use Committee (protocol #: AUP-2016-04-8684-2). Mice were housed with same sex littermates in groups of 2-5 animals per cage with ad libitum access to food and water, except where noted for specific behavior tests. Room temperature was set to 22 degrees C and humidity was not externally controlled. For most experiments, mice were housed on a regular 14h light/ 10h dark cycle (lights on at 6am). For the retrograde tracing experiments, behavior experiments, Grpr global knock-out mouse experiments, and Grpr-eGPP;A2A-Cre;Ai9 mouse experiments, mice were housed on a reverse 12h light/ 12h dark cycle (lights on at 9pm).<br><br>Both male and female mice were used across all experiments except for the global Grpr knock-out (KO) FISH experiments where male littermate WT and Grpr KO mice were used. Mice were postnatal day P30-P90 unless otherwise stated. The following mouse lines were used in this study: C57BL/6J, JAX strain #000664, Grpr::eGFP (GENSAT [MMRRC #036178-UCD]), Drd2-eGFP (GENSAT [MMRRC #000230-UNC]), Drd1-tdTomato (JAX strain #016204), CMV-Cre (JAX strain #006054), Grprfl (JAX strain # 033148), ChAT-IRES-Cre (JAX strain # 06410), and Ai9 (JAX strain # 07909). Grpr KO mice were generated by breeding CMV-Cre mice to Grprfl mice to knock out Grpr in all cells.

Wild animals	This study did not involve wild animals.
Reporting on sex	For most experiments, mice of both sexes were used. The only exception was for the global Grpr KO FISH experiments where male Grpr WT and Grpr KO mice were used.
Field-collected samples	There were no field-collected samples used.
Ethics oversight	Animal experiments were performed in accordance with protocols approved by the University of California, Berkeley, Institutional Animal Care and Use Committee (protocol #: AUP-2016-04-8684-2).

Note that full information on the approval of the study protocol must also be provided in the manuscript.

Plants

Seed stocks	*Report on the source of all seed stocks or other plant material used. If applicable, state the seed stock centre and catalogue number. If plant specimens were collected from the field, describe the collection location, date and sampling procedures.*
Novel plant genotypes	*Describe the methods by which all novel plant genotypes were produced. This includes those generated by transgenic approaches, gene editing, chemical/radiation-based mutagenesis and hybridization. For transgenic lines, describe the transformation method, the number of independent lines analyzed and the generation upon which experiments were performed. For gene-edited lines, describe the editor used, the endogenous sequence targeted for editing, the targeting guide RNA sequence (if applicable) and how the editor was applied.*
Authentication	*Describe any authentication procedures for each seed stock used or novel genotype generated. Describe any experiments used to assess the effect of a mutation and, where applicable, how potential secondary effects (e.g. second site T-DNA insertions, mosiacism, off-target gene editing) were examined.*
